# Data mining and machine learning approaches for the integration of genome-wide association and methylation data: methodology and main conclusions from GAW20

**DOI:** 10.1186/s12863-018-0646-3

**Published:** 2018-09-17

**Authors:** Burcu Darst, Corinne D. Engelman, Ye Tian, Justo Lorenzo Bermejo

**Affiliations:** 1 Department of Population Health Sciences, School of Medicine and Public Health, University of Wisconsin, 610 Walnut St. 1007 WARF, Madison, WI 53726 USA; 2 Department of Biochemistry and Medical Genetics, University of Manitoba, 745 Bannatyne Ave, Winnipeg, MB R3E 0J9 Canada; 3 Department of Electrical and Computer Engineering, University of Manitoba, 745 Bannatyne Ave, Winnipeg, MB R3E 0J9 Canada; 4 Institute of Medical Biometry and Informatics, University of Heidelberg, Im Neuenheimer Feld 130.3, 69120 Heidelberg, Germany

**Keywords:** Data mining, Machine learning, Genome-wide association study, Epigenome-wide association study

## Abstract

**Background:**

Multiple layers of genetic and epigenetic variability are being simultaneously explored in an increasing number of health studies. We summarize here different approaches applied in the Data Mining and Machine Learning group at the GAW20 to integrate genome-wide genotype and methylation array data.

**Results:**

We provide a non-intimidating introduction to some frequently used methods to investigate high-dimensional molecular data and compare the different approaches tried by group members: random forest, deep learning, cluster analysis, mixed models, and gene-set enrichment analysis. Group contributions were quite heterogeneous regarding investigated data sets (real vs simulated), conducted data quality control and assessed phenotypes (eg, metabolic syndrome vs relative differences of log-transformed triglyceride concentrations before and after fenofibrate treatment). However, some common technical issues were detected, leading to practical recommendations.

**Conclusions:**

Different sources of correlation were identified by group members, including population stratification, family structure, batch effects, linkage disequilibrium and correlation of methylation values at neighboring cytosine-phosphate-guanine (CpG) sites, and the majority of applied approaches were able to take into account identified correlation structures. The ability to efficiently deal with high-dimensional omics data, and the model free nature of the approaches that did not require detailed model specifications were clearly recognized as the main strengths of applied methods. A limitation of random forest is its sensitivity to highly correlated variables. The parameter setup and the interpretation of results from deep learning methods, in particular deep neural networks, can be extremely challenging. Cluster analysis and mixed models may need some predimension reduction based on existing literature, data filtering, and supplementary statistical methods, and gene-set enrichment analysis requires biological insight.

## Background

The GAW is held every 2 years. In advance of the workshop, real and simulated data sets are distributed worldwide. GAW participants analyze the data sets using different statistical techniques, compare their methods and results during a meeting in person, and describe major findings in individual and summary articles. The GAW20 was held in San Diego, California on March 4–8, 2017. The distributed real data set included phenotypic information on triglyceride levels and metabolic syndrome before and after treatment with fenofibrate, as well as epigenome-wide methylation and genome-wide genotype data. Simulated data was generated based on the structure of real data and also included phenotype, methylation, and genotype information. Individual contributions to the GAW20 were categorized by the Advisory Committee into 7 different groups. This article summarizes the methods and results from the Data Mining and Machine Learning group.

On the first day of the workshop, group members briefly presented their own contributions. Engaging discussions and intensive team work during 4 group meetings and several poster sessions resulted in a consensus summary of group findings, which was presented to all GAW20 participants during a plenary session.

**Fig. 1:**
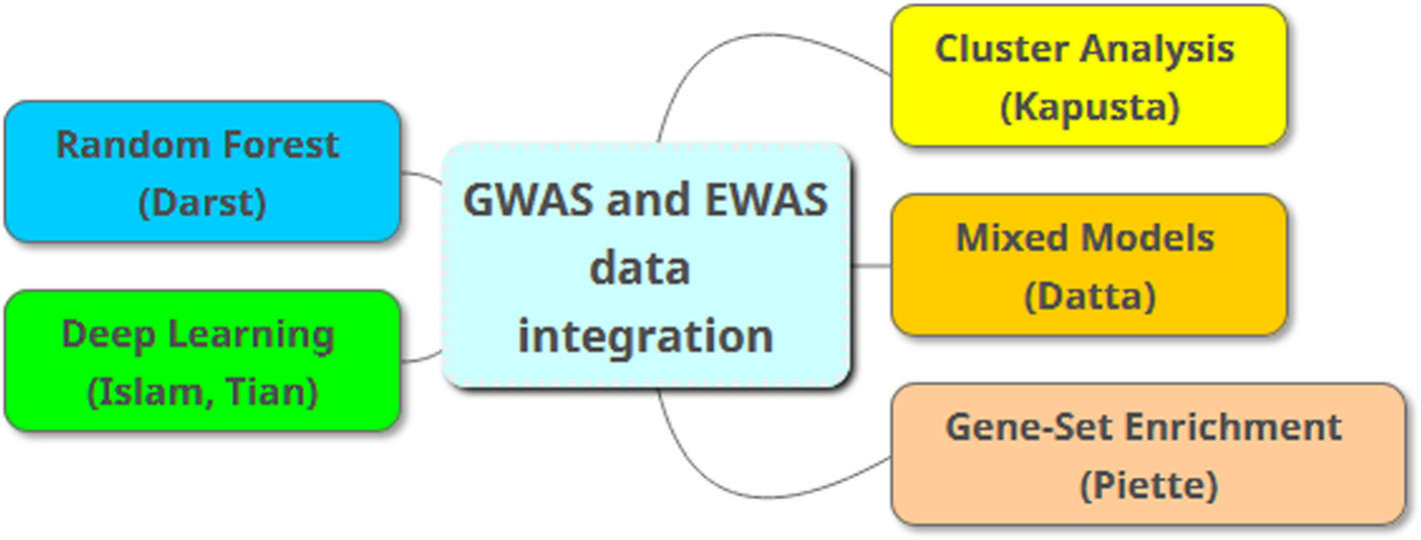
Mind-map with the 5 contributions from the Data Mining and Machine Learning group. The figure shows the main approach used by each group member. For example, Islam et al. used support vector machines in addition to deep learning, and Kapusta et al. applied random forest based on results from cluster analysis. EWAS, epigenome-wide association study; GWAS, genome-wide association study

Figure 1 represents a mind-map of the main approaches applied by members of the Data Mining and Machine Learning group. Darst et al. used recursive feature elimination in random forest to account for correlated high-dimensional data. Islam et al. aimed to predict triglyceride concentrations using deep learning, in particular a deep neural network, and compared the predictive ability of this approach with that of support vector machines. Kapusta et al. applied cluster analysis followed by random forest to identify groups of patients with similar global methylation patterns after fenofibrate treatment. Datta et al. proposed a 2-stage strategy (a variable screening for variable selection, followed by multiple regression) to circumvent high-dimensionality when fitting mixed effect models. Piette and Moore conducted gene-set enrichment analyses to infer potential biological function underlying drug response. After submission and peer review, 4 individual papers from the Data Mining and Machine Learning group were accepted for publication in the GAW20 proceedings, but we summarize here findings from the complete group [1–4].

Multiple layers of genetic and epigenetic variability are being simultaneously explored in an increasing number of health studies. Although the goals of each group within the Data Mining and Machine Learning group were quite heterogeneous, the integration of genome-wide genotype and methylation array data was a common theme. We provide a non-intimidating introduction to some methods that are frequently used to investigate high-dimensional molecular data and compare the different approaches used by group members. Common technical issues were detected, leading to practical recommendations for the integration of omics data.

## Methods

### Material

Table 1 shows the types of high-dimensional omics data and the phenotypes investigated by group members. Data were based on the Genetics of Lipid Lowering Drugs and Diet Network (GOLDN) study (see https://dsgwebwp.wustl.edu/goldn and accompanying publications in *BMC Genetics* for a detailed description of the design of the GOLDN study). Three of 5 contributions included data from 680 individuals with complete information on triglyceride levels before and after treatment, epigenome-wide methylation, and genome-wide genotype data. Two contributions investigated real data sets and simulated data was examined in 3 contributions. Except for Islam et al. who exclusively considered methylation data, all of the other groups examined both genome-wide genotype and epigenome-wide methylation data. Group contributions were quite heterogeneous regarding investigated phenotypes. All five contributions analyzed triglyceride concentrations, but they relied on different concentration measurements: original versus log-transformed concentrations, relative versus absolute concentration differences, or the concentration ratio before and after fenofibrate treatment.

**Table 1:** Investigated data in contributions from the Data Mining and Machine Learning group at the GAW20

Contribution	Sample size	Real data	Simulated data	GWAS	EWAS	Investigated phenotype(s)
Random forest (Darst)	680		X	X	X	log average post-TG − log average pre-TG
Deep learning (Islam)	993/499^a^	X			X	pre-TG, post-TG
Cluster analysis (Kapusta)	446	X		X	X	relative TG difference, metabolic syndrome
Mixed models (Datta)	680		X	X	X	post TG-pre TG
Gene-set enrichment (Piette)	680		X	X	X	log average post TG/log average pre TG

*EWAS* epigenome-wide association study, *GWAS* genome-wide association study, *TG* triglyceride concentration

^a^There were 993 participants in the Genetics of Lipid Lowering Drugs and Diet Network (GOLDN) study, but posttreatment methylation data was only available for 499

The following sections provide a hands-on introduction to 3 methods frequently applied to analyze high-dimensional omics data: random forest, deep learning, and support vector machines. We also briefly describe the use of the 3 methods by group members to integrate genotype and methylation data. We refer to the original publications in *BMC Proceedings* for information on mixed models and gene-set enrichment analysis, and for further technical details on the application of random forest, deep learning, and support vector machines.

### Random forest

Random forest is a data mining and machine learning method that has been applied to guide the integration of multi-omics data. Random forest relies on a multitude of decision trees, each built from a random subset of observation and features (here genome-wide genotypes and methylation values), to determine the importance of single features and the overall prediction ability of the model [5]. The use of trees allows for nonlinear relationships among features, making the approach particularly attractive for the identification of interactions between omics data. This was particularly suitable for the GAW20 simulation data, which included interactions between genetic variants and methylation sites.

The article by Acharjee et al. is an excellent example of the application of random forest to guide the integration of genomics, proteomics, and metabolomics data, to subsequently predict phenotypic traits [6]. The authors first used random forest to select the strongest predictors within each omics data set, which were then integrated into a regularized partial correlation network. It is important to remark here that Acharjee et al. used random forest to determine which omic features should be included in the integrative analysis, but not for the actual integration itself.

**Fig. 2:**
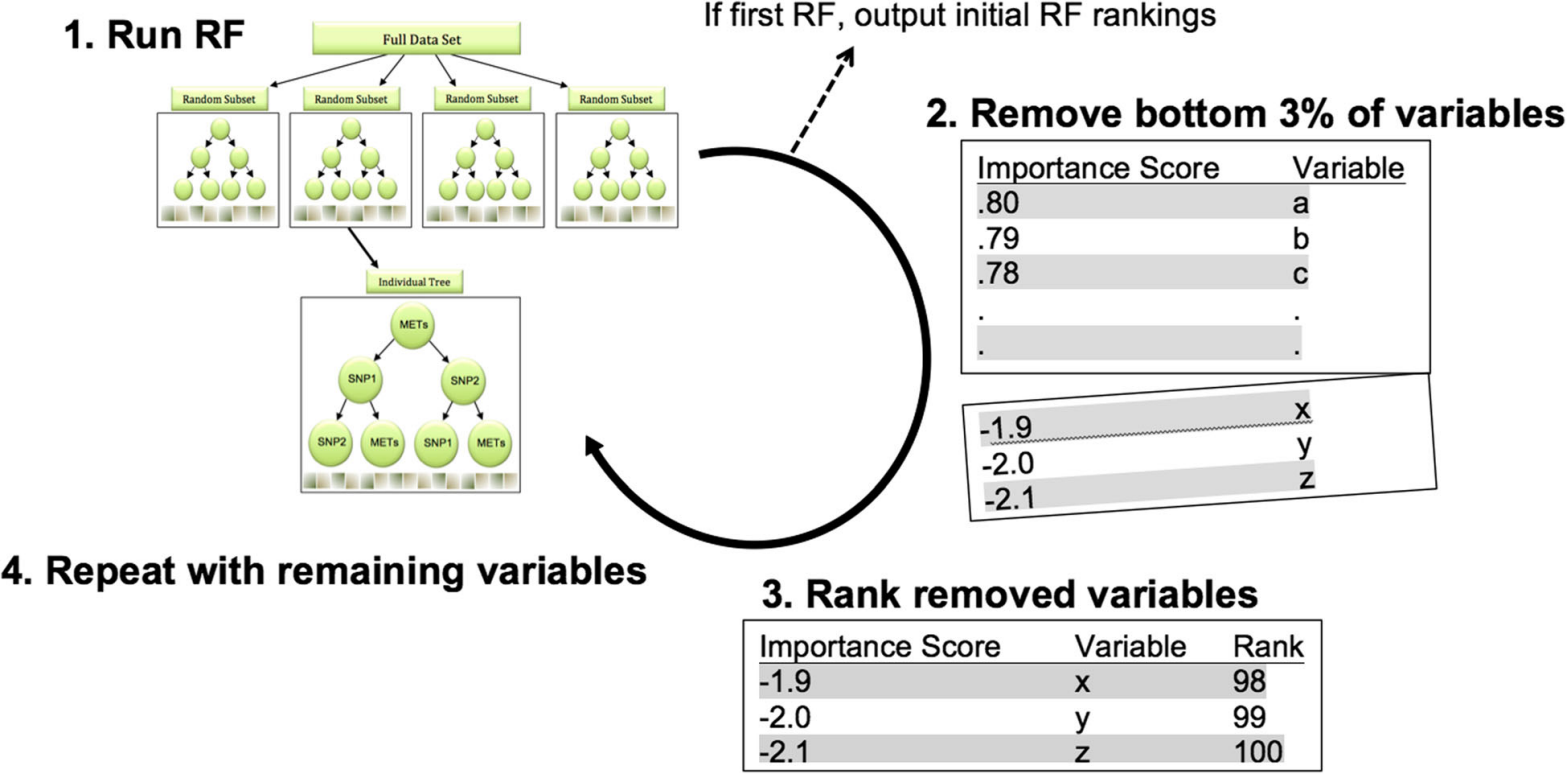
Recursive feature elimination–random forest applied to combined genome-wide genotype and methylation data. Recursive feature elimination was applied to random forest (RF) and consisted of the following steps: **a** running the random forest model; **b** removing features that random forest ranked in the bottom 3%; **c** ranking removed features starting with the lowest rank; and (**d**) recursively iterating until no additional features could be removed from the model. The comparison between random forest and random forest with recursive feature elimination relied on the full set of ranks

In the Data Mining and Machine Learning group, Darst et al. used random forest for the actual integration of simulated multi-omics data. They concatenated genome-wide genotyping and epigenome-wide methylation data provided by GAW20, and applied the R package “ranger” implementation of random forest to identify potential associations with triglyceride levels. Each random forest included 8000 trees, and each node considered a random 10% of the total number of features in the model for splitting (see Gregorutti et al. [7] for further parameter details). A major limitation of random forest is that multicollinearity strongly influences the performance of this approach. Recursive feature elimination was applied to mitigate this problem [7]. At the GAW20, Darst et al. used random forest to investigate highly correlated features, and applied recursive feature elimination to assess whether this alleviates the multicollinearity problem with high-dimensional omics data (Fig. 2).

Kapusta et al. analyzed real data by combining cluster analysis with an alternative random forest approach. The authors first reviewed the literature to select biologically relevant genotypes and methylation cytosine-phosphate-guanine (CpG) sites. Unsupervised clustering of individuals was then performed based on methylation data, which resulted in 3 separated clusters. Random forest was then applied to each cluster separately, integrating the selected genotypes and methylation sites.

### Deep learning with a deep neural network

**Fig. 3:**
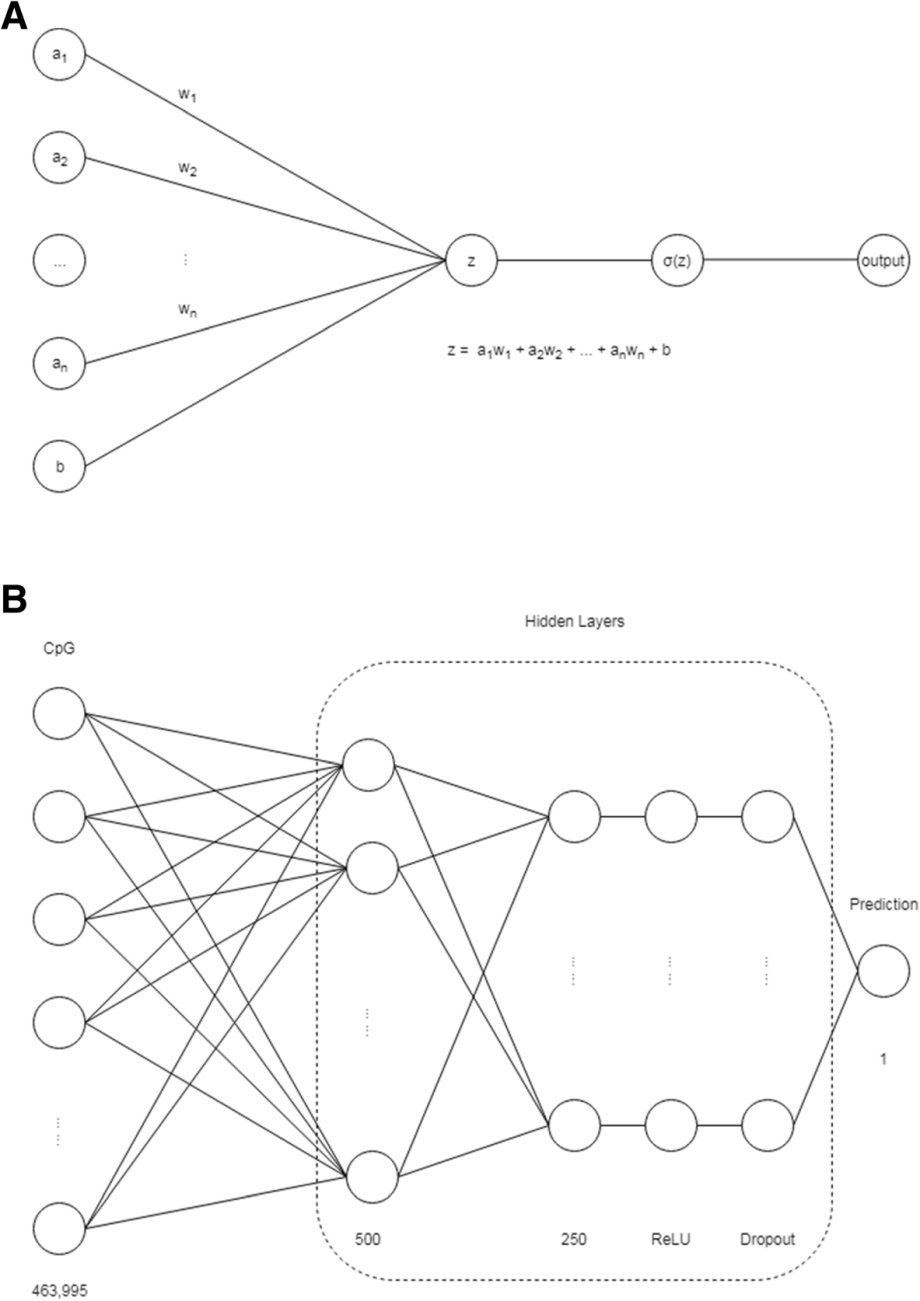
Deep learning model applied to genome-wide methylation data. Panel **a** represents an interconnected node (neuron), the basic element of artificial neural networks. *a*_*n*_ represents the *n*^*th*^ input signal into the neuron; *w*_*n*_ represents the corresponding weight of *a*_*n*_; and *b* is a random bias added to avoid overfitting. The sum of multiplied input values and random bias z is transformed into an output value by a fixed activation function σ. Panel **b** shows the specific deep neural network model used to investigate GAW20 methylation data. The first layer (input layer) included all 463,995 CpG sites. The second and third hidden layers were configured to 500 and 250 nodes, respectively. The fourth layer (ReLu) aims to nonlinearity, and the fifth layer (Dropout) targets at overcoming overfitting

Artificial neural networks are mathematical models that resemble neural networks in the nervous system. Artificial neural networks consist of interconnected nodes, called neurons (Fig. 3, panel a). At each neuron, input values (here epigenome-wide methylation values or output values from previous neurons) are multiplied by particular weights, a random bias is added to avoid model overfitting, and the sum of multiplied input values and random bias is transformed into an output value by a fixed activation function [8].

The basic layout of an Artificial Neural Network includes an input layer, single or multiple hidden layers, and an output layer (Fig. 3, panel b). The denomination *deep neural network* refers to an artificial neural network with multiple hidden layers. Deep neural networks take advantage of complex interconnection structures to extract high-level information from raw data. The user specifies the parameter setup of the overall network structure (eg, number of layers, node interconnections, bias distribution, and type of activation functions). Different methods (eg, back propagation) can be used to “train” a deep neural network; that is, to calculate the optimal weights that minimize differences between observed and final output values (here triglyceride concentrations).

In the Data Mining and Machine Learning group, Islam et al. proposed a deep neural network to predict individual triglyceride concentrations based on more than 450,000 epigenome-wide methylation values (see Fig. 3, panel b). The deep neural network was developed in C++ with the deep learning software framework *Caffe* [9]. Because of the complex nonlinear relationship between triglyceride concentrations and methylation, the authors used rectified linear units after 2 previous, fully connected hidden layers. The output of a rectified linear unit layer is a nonlinear transformation of the input according to the formula:

$$ f(x)=\left\{\begin{array}{c}x,x\ge 0\\ {}0,x<0\end{array}\right. $$

where *x* represents the input to a neuron.

To avoid overfitting, Islam et al. used a regularization technique denominated *dropout*, which randomly drops out hidden neurons from the hidden Dropout layer [10]. The trained model with fixed weights for each neuron could be used for future prediction. Technical details on the approaches used by the authors to train the proposed deep neural network can be found in the original *BMC Proceedings* publication.

### Support vector machines

Support vector machines is another data mining and machine learning method that has been used in omics integration studies [11]. A support vector machine is an algorithm that predicts a particular outcome (here triglyceride concentrations) by constructing a hyperplane, which maximizes outcome differences between sets of observations based on model features (here epigenome-wide methylation values). When observations cannot be linearly separated by a hyperplane, a kernel function can be applied to project the data into a higher dimension that is linearly separable. Once heterogeneous omics data sets are transformed using kernel functions, they are simple to combine, making support vector machines a good option for integrated omics analyses.

Lanckriet et al. used support vector machines to demonstrate that integrated primary protein sequence, protein–protein interaction, and messenger RNA expression data may result in higher prediction accuracy than separated types of data [12]. Recently, researchers proposed metaanalytic support vector machines that integrate omics data across multiple studies, allowing a metaanalysis of multi-omics data sets [13]. This novel method will facilitate the integration of studies with much larger sample sizes and likely ease the use of support vector machines for integrated analyses.

An important limitation of support vector machines shared by the majority of machine learning approaches is that, the larger the number of features relative to observations, the higher the probability that the model overfits the data [14]. Taking this limitation into consideration, Madhavan et al. combined multiple machine learning approaches to integrate gene expression, micro-RNA expression, copy number variant, and serum and urine metabolomics data. They first prefiltered using univariate analyses followed by support vector machines to avoid overfitting [15]. Top features were then integrated using random forest and network analysis.

In the Data Mining and Machine Learning group, Islam et al. compared the abilities of deep neural networks and support vector machines to predict individual triglyceride concentrations based on epigenome-wide methylation data.

## Results

To constrict the potential overfitting of applied machine learning methods, the majority of group members applied filters to genome-wide genotype and methylation data in advance to using their approaches (Table 2). Darst et al. constituted an exception as they did not use filters prior to the application of random forest to integrate omics data. Darst et al. were not able to explicitly identify simulated interactions between genotypes and methylation values affecting triglyceride levels, but were able to identify simulated associations between genotypes and triglyceride levels, which were dependent on interactions with methylation values. High-dimensionality is particularly an issue for support vector machines, and Islam et al. filtered epigenome-wide methylation values according to methylation variability (fixed thresholds for the difference between 90th and 10th methylation percentiles for each CpG site). When the authors selected hundreds of CpG sites with the highest variability instead of using the more than 450,000 original methylation signals, they found better prediction performances for deep neural networks than for support vector machines. Kapusta et al. reviewed the genome-wide association studies literature on metabolic syndrome and fenofibrate treatment, and combined principal component analysis, cluster analysis, and random forest to reduce dimensionality. The mixed model modification proposed by Datta et al. incorporates filtering through a first-stage screening based on 1 genetic variant at a time. For the selection of genes investigated in gene-set enrichment analyses, Piette and Moore applied paired t-tests for pre- and posttreatment methylation levels, and they fitted linear models with phenotype as response, and methylation as explanatory variables, in advance to the web-based gene set analysis toolkit (see http://webgestalt.org/option.php).

**Table 2:** Applied filters, possibility of adjustment for familial correlation, and strengths and limitations of applied methods in the contributions from the Data Mining and Machine Learning group at the GAW20

Contribution	Applied filters	Potential correlation adjustment	Strengths	Limitations
Random forest (Darst)	None	Yes	Model free; adequate for high-dimensional data	Does not work well with highly correlated variables
Deep learning (Islam)	Methylation variability	Yes	Robust; adequate for high-dimensional data	Difficult result interpretation, tough parameter set up, large sample sizes are needed
Cluster analysis (Kapusta)	Reported genome-wide association studies on metabolic syndrome and fenofibrate treatment, principal component analysis, random forest	Yes	Intuitive cluster interpretation	Previous dimension reduction can be indicated
Mixed models (Datta)	Mixed models modification	Yes	Simple regression framework	Not indicated for low-dimensional data
Gene-set enrichment (Piette)	T-tests and linear regression	No	Circumvents multiple testing	Requires biological insight

Different sources of correlation were identified by group members, including population stratification, family structure, batch effects, linkage disequilibrium and correlation of methylation values at neighboring CpG sites. Although the majority of applied approaches are able to take into account identified correlation structures (see Table 2), familial correlation was only taken into account by Kapusta et al. through family identifiers in cluster analyses, and by Datta et al. through the kinship matrix, which was computed with the INBREED procedure of SAS version 9.4 and subsequently taken into account using the MIXED procedure. Other group members did not adjust for familial correlation either because family structure was not explicitly accounted for in data simulations or because the applied approach could not account for correlation (gene-set enrichment analysis).

## Discussion

Engaging discussions at the GAW20 motivated us to formulate some practical recommendations when designing an integrative analysis of omics data. Table 2 summarizes the strengths and limitations of the data mining and machine learning approaches applied by group members. The ability to efficiently deal with high-dimensional omics data, and the model-free nature of the approaches that did not require detailed model specifications were clearly recognized as the main strengths of applied methods. Darst et al. found that the application of recursive feature elimination to Random Forest did not appear to improve the performance of Random Forest in the presence of multicollinearity, suggesting that this approach may not scale to large multi-omics data sets.

Islam et al. reported that the parameter setup and the interpretation of results from deep learning methods, in particular deep neural networks, can be extremely challenging. Their results from deep learning models revealed that DNA methylation profiles measured at pretreatment are able to predict posttreatment triglyceride concentrations more accurately than DNA methylation profiles measured at posttreatment, suggesting a long-term epigenetic effect on phenotypic traits. In contrast to the majority of studies that use deep learning approaches for classification, Islam et al. explored at the GAW20 the potential of deep learning models within a regression analysis framework, and they demonstrated that the method generally works well for high-dimensional epigenomic data. Cluster analysis and mixed models may benefit from some predimension reduction based on existing literature, data filtering, and complementary statistical methods. Gene-set enrichment analysis requires biological insight, which is growing at a fast pace.

## Conclusions

Genetic studies are increasingly exploring multiple layers of biological variability, which may range from inherited traits to proteomics and exposomics. Members of the Data Mining and Machine Learning group applied some of the most common data integration approaches at the GAW20. Although investigated data sets and applied approaches were quite different, some practical recommendations can be formulated based on group findings. For high-dimensional data, it can be advantageous to use methods that do not require accurate model specifications (model-free approaches), for instance, many machine learning methods. But this advantage often comes with the difficulty of interpreting the structure and parameters of the final model used for prediction. Complex correlation structures are standard in omics data, and the majority of machine learning approaches generally are able to take observational correlation into account. However, highly correlated features among high-dimensional data may still pose a challenge and additional methodological research is needed to bypass the problem. Most members of the Data Mining and Machine Learning group addressed this by filtering the omics data prior to integrating, but reducing the sensitivity of results to highly correlated data would also be appropriate.

## Abbreviations

CpG: Cytosine-phosphate-guanine
GAW: Genetic Analysis Workshop
GOLDN: Genetics of Lipid Lowering Drugs and Diet Network
